# Beyond reproductive rights: implementing the Africentric reproductive justice framework in sexual and reproductive health and rights litigations in Africa

**DOI:** 10.1080/26410397.2025.2570579

**Published:** 2025-10-10

**Authors:** Moses Mulumba, Jessica Oga, Nimrod Muhumuza

**Affiliations:** a Director General, Afya na Haki, Kampala, Uganda; b Head of Regionalism, Afya na Haki, Kampala, Uganda.; c Head of Research, Afya na Haki, Kampala, Uganda

**Keywords:** Africentric framework, colonial legacies, intersectionality, litigation, reproductive justice, sexual and reproductive health and rights

## Abstract

This paper analyses emerging trends in judicial approaches to sexual and reproductive health and rights (SRHR) in Africa, arguing for a paradigm shift from the conventional rights-based framework that emphasises “choice” to an Africentric Reproductive Justice framework (ARJ). It employs a qualitative research design, combining a literature review with an in-depth analysis of eight purposively selected SRHR court cases from African countries and the United States to examine how recent judicial decisions reflect a growing integration of reproductive rights with broader social justice issues. The proposed ARJ framework is grounded in the reproductive justice theory, incorporating conceptual principles of decoloniality and Africentrism, which emphasises amplifying positive African narratives, leveraging African-based judicial institutions, involving civil society organisations, and empowering African individuals to engage with judicial systems for reproductive justice. This approach addresses intersectionality, race, class, gender, and socioeconomic status in SRHR issues in Africa. The findings demonstrate that African courts are increasingly balancing competing rights and interests in culturally sensitive ways, reflecting an evolving understanding of reproductive justice in African contexts. This paper concludes by arguing that achieving genuine reproductive justice necessitates moving beyond the “right to choice”. It requires deconstructing enduring structural barriers and addressing historical injustices perpetuated by colonial legacies, and the ARJ framework, rooted in decolonial principles, presents a holistic, context-specific approach towards achieving equitable SRHR for all individuals on the continent.

## Plain language summary

This paper examines trends in judicial approaches to sexual and reproductive health and rights (SRHR) in Africa. It calls for a shift from the usual rights-based framework that focuses on “choice” to an Africentric Reproductive Justice framework (ARJ). The research uses qualitative methods, combining a literature review with an analysis of eight selected SRHR court cases from both African countries and the United States. This approach helps to explore how recent court decisions link reproductive rights to wider social justice issues. The ARJ framework is rooted in reproductive justice theory. It incorporates ideas of decoloniality and Africentrism, highlighting positive African narratives. It also advocates for the use of African judicial institutions, the involvement of civil society organisations, and the empowerment of individuals to pursue reproductive justice. This method addresses intersectionality, race, class, gender, and socioeconomic status in SRHR issues across Africa. The findings show that African courts are increasingly balancing competing rights and interests in ways that respect cultural contexts. This indicates a growing understanding of reproductive justice in African settings. The paper concludes that achieving true reproductive justice means going beyond the “right to choice”. It requires confronting structural barriers and historical injustices from colonial times. The ARJ framework offers a comprehensive, context-specific approach to ensuring equitable SRHR for everyone on the continent.

## Résumé

Cet article analyse les tendances émergentes dans les approches judiciaires de la santé et des droits sexuels et reproductifs en Afrique, plaidant en faveur d’un changement de paradigme pour passer du cadre conventionnel fondé sur les droits, qui privilégie le « choix », à un cadre de justice reproductive afrocentrique (ARI). Il utilise une conception de recherche qualitative, combinant un examen des publications et une analyse approfondie de huit affaires judiciaires en matière de santé et de droits sexuels et reproductifs sélectionnées à dessein dans des pays africains et aux États-Unis, pour établir comment les récentes décisions judiciaires traduisent une intégration croissante des droits reproductifs dans les questions plus larges de justice sociale. Le cadre ARI proposé est basé sur la théorie de la justice reproductive, intégrant des principes conceptuels de décolonialité et d’afrocentrisme, qui souhaite amplifier les discours africains positifs en s’appuyant sur les institutions judiciaires basées en Afrique, en faisant participer les organisations de la société civile et en donnant aux Africains les moyens d’interagir avec les systèmes judiciaires en matière de justice reproductive. Cette approche traite l’intersectionnalité, la race, la classe sociale, le genre et le statut socio-économique dans les questions de santé et de droits sexuels et reproductifs en Afrique. Les résultats montrent que les tribunaux africains équilibrent de plus en plus les droits et les intérêts concurrents de manières culturellement sensibles, ce qui reflète une évolution de la compréhension de la justice reproductive dans les contextes africains. L’article conclut en affirmant que pour parvenir à une véritable justice reproductive, il est nécessaire d’aller au-delà du « droit de choisir ». Cela exige de déconstruire les obstacles structurels persistants et de s’attaquer aux injustices historiques perpétuées par l’héritage colonial. Le cadre ARI, ancré dans les principes décoloniaux, représente une approche holistique et contextuelle en vue de parvenir à l’équité en matière de santé et de droits sexuels et reproductifs pour tous les habitants du continent.

## Resumen

Este artículo analiza las tendencias emergentes en los enfoques judiciales relativos a la salud y los derechos sexuales y reproductivos (SDSR) en África, y argumenta a favour de cambiar el paradigma del marco convencional basado en los derechos que pone énfasis en “la elección” por el Marco de Justicia Reproductiva Afrocéntrica (JRA). Emplea el diseño de investigación cualitativa, combinando una revisión de la literatura con un análisis a fondo de ocho casos judiciales sobre SDSR seleccionados intencionalmente de países africanos y Estados Unidos, con el fin de examinar cómo decisiones judiciales recientes reflejan la creciente integración de los derechos reproductivos en asuntos de justicia social más amplios. El marco JRA propuesto está fundamentado en la teoría de justicia reproductiva e incorpora los principios conceptuales de la decolonialidad y el Afrocentrismo, que hace hincapié en amplificar narrativas africanas positivas, aprovechando las instituciones judiciales con sede en África, involucrando a organizaciones de la sociedad civil y empoderando a las personas africanas para interactuar con los sistemas judiciales a fin de lograr la justicia reproductiva. Este enfoque aborda interseccionalidad, raza, clase, género y condición socioeconómica en asuntos de SDSR en África. Los hallazgos demuestran que las cortes africanas cada vez más establecen un equilibrio entre derechos e intereses en conflicto de maneras culturalmente sensibles, lo que refleja una comprensión en evolución de la justicia reproductiva en contextos africanos. Este artículo concluye argumentando que para lograr la justicia reproductiva genuina es necesario trascender el “derecho a la elección”. Es necesario deconstruir las barreras estructurales duraderas y abordar las injusticias históricas perpetuadas por legados coloniales, y el marco JRA arraigado en los principios decoloniales presenta un enfoque holístico relacionado específicamente con el contexto para lograr SDSR equitativos para todas las personas en el continente.

## Introduction

Reproductive rights have been defined as “the right to decide freely and responsibly the number, spacing, and timing of children”, to achieve the highest standard of sexual and reproductive health, and to make informed reproductive decisions free from discrimination, coercion, and violence.^1^ In 1994, the International Conference on Population and Development (lCPD) in Cairo broke new ground by linking reproductive rights to human rights.^2^ This marked a significant step forward in advancing the reproductive rights agenda and ensuring that women’s reproductive choices were protected as fundamental human rights. However, it is crucial to recognise that the human rights paradigm in light of sexual and reproductive health and rights (SRHR) is not free of criticism.^3^ For all of the progress it has registered since it acquired near-universal status, it should be recognised, as Ngwena does, that the foundation of the modern human rights framework is clothed in Western imperialism and colonialism (p. 13).^3^ While efforts have been made to mitigate the colonial origins and impact of the human rights paradigm, such as through the adoption of regional instruments, these criticisms remain.^4^ Herr, for instance, argues that an uncritical embrace of the Women’s Rights as Human Rights movement, despite its Eurocentric provenance, could potentially further perpetuate “the imperialist stance of the colonial era and erode culturally diverse modes of gender justice in the Global South”.^5^ The reproductive rights framework, itself grounded in human rights, is at once implicated by the colonial and imperial origins of the latter. Pizzarossa traces the evolution of the SRHR movement from its eugenic and racist roots to its capstone in Cairo at the ICPD in 1994.^6^ Tellingly, prior to the ICPD, SRHR issues had been framed in the language of population control, and what Kuumba describes as reproductive imperialism.^7^ The ICPD changed the conversation around population issues from pure population control through family planning advocated for in the Global South, through the “population establishment”, to broader issues related to reproductive freedom and safe sex.^6,8^ Consequently, uncritically anchoring reproductive rights in such a framework is a failure to recognise and account for the experiences of those whom colonial powers have historically deemed subaltern. The progenitors of the modern reproductive justice movement – Black women and women of colour, for instance – criticised the approach at the ICPD, arguing that the then-prevailing reproductive rights framework failed to adequately address their unique experiences, challenges, and needs.^9^ In response, the reproductive justice framework emerged as a more inclusive and more encompassing reproductive justice that seeks to achieve the “complete physical, mental, spiritual, political, social and economic well-being of women and girls”.^10^ The reproductive justice movement nonetheless relies partly on the human rights framework, including the International Bill of Rights, to hold states accountable for acts or omissions that are inimical to the realisation of full reproductive autonomy, especially in Africa.^10^

The 1973 landmark decision of *Roe v Wade*^11^ disguised the complex ways in which laws, policies, and public authorities punished or rewarded women’s reproductive activity.^12^ In purporting to treat all women equally using the rights framework, particularly the right to privacy, it masked the different ways in which systemic and institutional racism and the legacy of enslavement combined to deny Black women and women of colour actual access to reproductive freedom and autonomy, despite their claim to formal equality.^13^ As the classic exemplar of the potential and limits of the rights framework, *Roe* promoted a hands-off approach to state regulation of reproductive rights. The government was not to interfere in the decision-making or “choice” of an individual on whether to terminate or keep a pregnancy.^14^ However, this approach assumed the existence of several choices emanating from access to resources, which was not the case for many women of colour. As one activist has assessed, “to be pro-choice, you must have the privilege of having choices”.^15^ Thus, it is vital to put forward a framework that goes beyond merely focusing on “choice” to address the socioeconomic impact of the right to have or not to have a child. The reason is not far-fetched; Africa’s struggle with SRHR is characterised by unique challenges^16–18^ like poverty, patriarchy, environmental degradation, economic disparities, inadequate health infrastructure, political instability, economic volatility and restrictive legal and policy frameworks. An inordinate focus on the law and legal restrictions without incorporating the other systemic barriers to reproductive autonomy can only lead to a truncated assessment of the problem and solution.^19^

Thus, the reproductive justice framework acknowledges that true reproductive justice can only be realised when intersecting issues that affect an individual’s life are addressed. It argues that economic hurdles, such as the costs of birth control, pregnancy, childbirth, and postnatal care, limit a person’s freedom to make informed reproductive choices.^20^ It demands that women and girls have the agency and resources needed to make autonomous choices over their bodies, sexuality, and reproductive lives.^21^ Reproductive justice highlights that one cannot isolate abortion from other historical social justice issues that concern Black people and communities of colour.^22^ It therefore calls for the need to move from the hands-off, non-state interference, compartmentalised approach of reproductive rights undergirded by the liberal human rights tradition to a more inclusive understanding of the intersections between race, class, and reproductive autonomy in order to rectify historical injustices and foster true equity.

Therefore, in this paper, we employ a decolonial and Africentric framework to examine the limitations of the traditional rights-based approach and propose an alternative, context-specific strategy for advancing SRHR in Africa.

As exemplified by the adoption of the Maputo protocol, the traditional rights-based approach to SRHR has been instrumental in advancing reproductive autonomy. However, this approach has notably been limited in addressing the complex realities and historical injustices that shape the SRHR landscape in Africa, particularly concerning the broader social, economic, and political contexts that enable or constrain these choices. It is partly for this reason, for instance, that the Maputo Protocol’s article 17 on the right of women to live in a positive cultural environment has received scant attention compared to article 14 on reproductive rights and abortion. This is despite the fact that, in Africa at least, cultural stereotypes about women and girls have a major role in curtailing their reproductive rights and freedoms.^23^

In African countries, where legacies of colonialism, systemic inequality, and resource constraints intersect, we argue that a narrow focus on choice as understood in the context of *Roe* above may not suffice to ensure actual and equitable access to SRHR. We therefore argue that an Africentric Reproductive Justice (ARJ) framework, grounded in African perspectives, institutions, and solutions, offers a more comprehensive and context-specific approach to addressing SRHR challenges in Africa. Through the examination of decided SRHR cases across the continent, this paper demonstrates that a judicial approach that integrates reproductive rights with broader social justice concerns is critical. The findings presented in this paper thus highlight the need for a paradigm shift towards ARJ in SRHR litigation and policymaking.

## Methodology

This study employs a qualitative research methodology to analyse emerging judicial approaches to SRHR in Africa and to develop the arguments for the Africentric Reproductive Justice (ARJ) framework. The primary methods are a literature review and an analysis of judicial decisions on SRHR-related cases from selected African countries and other jurisdictions.

### Literature review

A comprehensive literature review was conducted to examine the existing scholarship on SRHR in Africa, including studies that explored the nuances of the conventional rights-based approach as well as those that embraced the broader theory of the reproductive justice framework. This review included scholarly studies, books, reports, and policy documents from various disciplines, including law, public health, women's studies, and African studies.

The literature review informed the study's theoretical and conceptual framework. The theoretical foundation is grounded in reproductive justice theory, which emphasises the intersection of reproductive rights with social, economic, and political inequalities.^24^ Conceptually, the study employs both decoloniality and Africentrism. Decoloniality, as defined by Mignolo,^25^ seeks to dismantle hierarchical structures of race, gender, heteropatriarchy, and class that continue to shape knowledge production and lived experiences in Africa. Africentrism positions Africa and its peoples as subjects of their own narrative, emphasising positive African narratives, the importance of African institutions, the role of African civil society organisations, and the potential of African individuals in advancing reproductive justice.^26^

This review identified significant gaps and limitations in the current choice and bodily autonomy approach to SRHR in Africa, highlighting the need for a more comprehensive, context-specific framework that addresses the unique historical, cultural, and socioeconomic factors influencing reproductive health outcomes on the continent.

This study did not involve human subjects research and, therefore, did not require ethics committee approval or informed consent.

### Reflexivity statement

The authorship team comprises three African researchers, all based in Kampala, Uganda, at Afya na Haki. The group brings together senior leadership and mid-career expertise in law, policy, and research on sexual and reproductive health and rights. In terms of gender and background, the team reflects both male and female perspectives, with professional experience spanning legal practice, health rights advocacy, and regional policy engagement. The collective positionality of the authors, as African scholars situated within the continent, informs the analysis and ensures that the arguments advanced are grounded in local realities, regional priorities, and Africentric approaches to reproductive justice.

### Conceptual framework: decoloniality, Africentrism and the invisible constitution

Leszczynski observes that there are extra-legal criteria that are a crucial part of reasoning that affect the process, results and roles of particular rules of judicial interpretation.^27^ These values include equity, good manners, good faith and social interest. Judges have also routinely implied principles such as federalism, responsible government, the separation of powers, and democratic representative government. In other words, guidance in constitutional interpretation is not confined to principles implied or expressed in the constitution but may also involve broader fundamental principles of liberty and justice. For instance, determining the constitutionality of Botswana’s anti-sodomy law, the country’s High Court approvingly cited Bentham’s utilitarian approach and the need to ensure “pleasure,” “good,” or “happiness” for all.^28^ In other words, the object of all legislation must be “the greatest happiness of the greatest number.”^28^ These values may also be moral, political or economic. The High Court of Botswana has unequivocally observed that the Constitution ought to be interpreted according to the imperatives of the prevailing socio-political context. Mere classic linguistic formalism is, thus, discouraged.^28^ It is somewhat surprising that very little literature examines the topic of extra-legal or extra-textual constitutional interpretation. One North American scholar sums up the controversy this way:

*“[I]n reviewing laws for constitutionality [or acts or omissions of public and private entities], should judges confine themselves to determining whether these laws [or acts and omissions] conflict with norms derived from the written Constitution? Or may they also enforce principles of liberty and justice when the normative content of these principles is not to be found within the four corners of [the] founding document?”^29^*

Courts in Pakistan, Uganda, Ghana, Nigeria, Zimbabwe, Seychelles, and Grenada have made consequential appeals to necessity with far-reaching detrimental impacts on their legal and constitutional order.^30^ Winterton admits that sometimes the boundary between constitutional rules and principles and extra-constitutional notions is indistinct because the constitutions often include “implied powers and prohibitions.”^31^ These applicable aids to interpretations may be implied in the constitution; if not in a specific provision, then, at least, in the constitutional structure or the “emanations” and “penumbras” of specific provisions or through the common law doctrine of *stare decisis*.^31^

Statutes and public acts or omissions cannot be understood or interpreted in a vacuum, especially when it comes to matters as controversial, fluid, and dynamic as SRHR. They must be placed in their moral, social, cultural, and political context, which requires judges to pay attention to more than just the black-letter law they are interpreting.^19^ This is why, for instance, the constitutional provisions establishing the judiciaries in Zimbabwe, Kenya and Uganda state that judicial power is derived from the people.^32–34^ Uganda’s Constitution further requires courts to exercise this judicial power in the name of the people and in conformity with the law and with the “values, norms and aspirations of the people.”^34^ The preamble to South Africa’s Constitution speaks of creating a society based on “democratic values, social justice, and fundamental rights”.*

These unwritten rules are now referred to as the invisible constitution – a complex superstructure of rules, doctrines, standards, legal tests, judicial precedents, legislative and executive practices, and cultural and social traditions or values. Their relevance to constitutional interpretation for adjudicating sexual and reproductive health, rights, and reproductive justice has not been the subject of scholarly analysis. Evidence shows that African judiciaries have utilised interpretative techniques that speak directly to the peculiar African experience(s) to determine SRHR cases.^35^ These techniques include evaluating socioeconomic and cultural factors and their impact on implementing SRHR, and it is these techniques that are assessed later on in the paper. It is through this invisible constitution that courts apply the principles of decoloniality and Africentrism.

### Decoloniality

This study is grounded in a decolonial framework, recognising that the financing, rhetorical, ideological, and theoretical bases of SRHR in Africa are often rooted in colonial legacies.^36^ Coloniality, in this case, refers to the enduring influence of colonisation on Africa’s SRHR landscape, shaping material, intellectual, emotional, and spiritual conditions.^37,38^ The colonial period disrupted and distorted indigenous SRHR practices, creating “long-standing power imbalances” that continue to “define culture, labour, interpersonal relations, and knowledge production” even after African nations gained political independence.^3^

The impact of these colonial legacies on SRHR in Africa is profound and multifaceted.^39^ Legal systems introduced by colonial powers continue to shape judicial practices, often prioritising Western notions of individual rights over traditional African communal values.^39^ This is evident in the persistence of conservative laws criminalising abortion and same-sex unions in many African countries, reflecting the imposition of Victorian-era morality on African societies.^40^

Colonial-era health systems, characterised by unequal resource allocation favouring European settlers, have led to enduring disparities in healthcare access.^41–43^ Urban areas, historically inhabited by colonisers, continue to have better facilities, while rural areas remain underserved.^44^ This inequitable distribution of health resources continues to significantly impact access to SRHR services, particularly for marginalised communities in Africa to this day.

These colonial legacies underscore the limitations of traditional rights-based approaches to SRHR in Africa. They highlight the need for a reproductive justice framework that goes beyond individual rights to address the complex interplay of power, culture, and history-shaping African reproductive experiences.

For this reason, this paper adopts Mignolo’s definition of ***decoloniality*** as “ways of thinking, knowing, being and doing that began with, but also precede the colonial enterprise and invasion.”^25^ Decoloniality offers a contextual, relational, and practice-based frame of reference that seeks to dismantle the hierarchical structures of race, gender, heteropatriarchy, and class that continue to control life, knowledge production, thought structures, and lived experiences on the continent.^3^ It centres research, methodologies, and practices within indigenous communities rather than relying on colonised institutions and epistemologies. In the context of SRHR, a decolonial approach demands the recognition and validation of African knowledge systems and practices, challenging the dominance of Western paradigms in reproductive health discourse and policy.

Judiciaries in Africa have tended to shy away from the need for a decolonial approach to statutory interpretation and adjudication. This is partly due to the colonial construction of African judiciaries – designed to have only a marginal interaction with the day-to-day experiences of their populace (p. 139).^37^ Judges have supplanted decoloniality with an appeal to Africa’s cultural and social mores and their impact as interpretative aids – part of the invisible constitution – as discussed in the selected cases. Having had their metaphorical umbilical cord severed at independence, African judiciaries in countries such as South Africa, Kenya, and Uganda have grown into mature adults who do not have to rely on precedent that is inimical to the conditions, realities and aspirations of the people they serve.^39^

### Africentrism

Integral to the decolonial framework within the African context, our study employs an Africentric framework, positioning Africa and its peoples as the subjects of their own story. It seeks to centre historical and ontological experiences and perspectives from the continent,^†^ drawing from African philosophies, wisdom, and value systems that reflect an underlying unity across diverse forms and practices. Africentrism proposes that people of African descent or cultural orientation should centre their view and evaluation of the world within their own historical and ontological framework.^26,45^ In the context of SRHR and reproductive justice, an Africentric approach emphasises the importance of drawing lessons from indigenous viewpoints and knowledge to inform the programming and advocacy of SRHR. It challenges us to abandon the top-down approach often employed by civil society organisations when engaging with communities – the supposed beneficiaries of their work.

Our understanding of Africentrism is four-pronged. It includes ^26,45^ an articulation of positive African narratives, assessing the role of African institutions, promoting the work of African organisations and broader civil society, and highlighting the contributions of African individuals to the cause.

The utility of narrative change from Africa is vital, given that the continent shoulders a disproportionate share of the world’s SRHR burden. Yet Africa is often portrayed as a continent perpetually in need of assistance and incapable of providing leadership to the rest of the world in matters of SRHR and reproductive justice.^46,47^ When internalised, these incomplete and harmful narratives risk negating any form of African agency and undermining the continent’s ability to define its own destiny, even within the SRHR space. An illustration of this denial of agency is the stupefying narrative that components of SRHR, such as safe and legal abortion, are un-African and, therefore, must be severely restricted on the continent. Positive narratives on this front should directly confront these false depictions by indicating that the termination of pregnancy was commonplace in many pre-colonial African societies and was only criminalised at the advent of colonialism.^48^ Consequently, an Africentric framework highlights Africa’s robust human rights architecture, which has the potential for guaranteeing SRHR and realising reproductive justice. Just like the rest of the world, Africa has a robust human rights architecture with immense potential for guaranteeing SRHR and realising reproductive justice. Indeed, the continent boasts of the only binding human rights instrument to guarantee reproductive rights, specifically the right to abortion in the Maputo Protocol. The Maputo Protocol’s monitoring body, the African Commission on Human and Peoples’ Rights continues to engage governments to ensure its full implementation, even though a lot more could be done. African institutions, particularly courts and quasi-judicial bodies at both national and regional levels, are playing an increasingly pivotal role in advancing reproductive justice. The willingness of bodies like the African Committee of Experts on the Rights and Welfare of the Child, East African Court of Justice and the ECOWAS Court of Justice^49^ to hear and determine SRHR-related cases demonstrates the continent's capacity to address these issues through its own judicial mechanisms to create autochthonous jurisprudence that is immune to the politics and shifts, particularly of the West. This institutional engagement is crucial for interpreting and enforcing SRHR within an African context.

As mentioned, Africentrism champions the work of African organisations and broader civil society that shape the landscape of reproductive justice in Africa. Indigenous and international civil society organisations like Afya na Haki^50^ and the Centre for Health, Human Rights and Development (CEHURD)^51^ have been instrumental in strategic litigation and advocacy for SRHR. Their work exemplifies African-led initiatives pushing for reproductive justice, grounded in local contexts and needs. It is hard to overstate organised civil society's role in realising reproductive justice on the continent. This role has not always been positive, as evidenced by the well-documented racist and colonial roots of major organisations that pushed a population control narrative at the dawn of decolonisation in Africa. Contemporary civil society is neither homogenous nor united in its goals to advance reproductive justice. In South Africa and Kenya, organised opposition has directly petitioned the court in its affront on reproductive rights and, so far, has been unsuccessful in the main.^52^ The long-term impact of their efforts is yet to be seen.

Individual African lawyers and activists also play crucial roles in advancing reproductive justice through litigation and advocacy. The contributions of experts like Onyema Afulukwe in Nigeria^53^ have been pivotal in challenging restrictive laws and policies, demonstrating the significant impact that individual actors can have on shaping the SRHR landscape across the continent. In certain instances, the bravery of these individuals has been particularly evident, as seen in the case of *Mildred Mapingure*,^54^ which is explored in greater detail later in this paper.

It is, however, crucial to recognise that invoking the rhetoric of “African values and African culture” in the SRHR space can sometimes be used to restrict access to necessary information, medical services, and products or to deny them to groups of people deemed unworthy. We reject this distorted and truncated interpretation of culture and history. Instead, we align with Tamale’s assertion that:

*“the African decolonisation/decolonial project is fundamentally about one thing: restoring the dignity of African people. By no means is it focused on a naive desire to return to a romanticised pre-colonial past. Rather, it is about reconstructing the relationship between African people and the colonisers”.^37^*

### Theoretical framework

This study is grounded in reproductive justice theory, which provides a comprehensive lens for analysing SRHR issues in Africa. Reproductive justice theory goes beyond the conventional reproductive rights framework to incorporate social justice and human rights principles.^20^ It is based on three core principles: the right to have a child, the right not to have a child and the right to parent children in safe and healthy environments.^55^

This theory recognises that reproductive rights are inextricably linked to social, political, and economic conditions.^2^ It emphasises the intersectionality of various forms of oppression, including racism, classism, and sexism, which impact reproductive health and decision-making.^2^

In the African context, the reproductive justice theory allows us to examine SRHR issues through a lens that acknowledges the complex historical, cultural, and socioeconomic factors unique to the continent. It provides a framework for understanding how colonial legacies, systemic inequalities, and cultural norms intersect to shape reproductive experiences and access to SRHR services.

By integrating reproductive justice theory with decolonial and Africentric approaches, this study advances a nuanced and contextually grounded understanding of SRHR in Africa. Moving beyond the conventional rights-based approach, it interrogates the structural and epistemic inequities rooted in coloniality that continue to shape reproductive experiences on the continent. Centring indigenous African cosmologies, lived realities, and expressions of agency, this approach emphasises holistic well-being over individualistic legal entitlements. This theoretical synthesis not only informs the analysis of judicial decisions but also underpins the development of the Africentric Reproductive Justice (ARJ) framework, offering a transformative lens through which SRHR can be reimagined and pursued in African contexts.

The ARJ framework is anchored in three interrelated pillars:

1. **Reclamation of African agency and narratives**: Prioritises the revitalisation of indigenous African knowledge systems, amplifies community-driven advocacy by civil society organisations (CSOs), and empowers African institutions and individuals to co-construct reproductive justice agendas that reflect localised epistemologies and aspirations.
2. **Decolonial epistemic resistance**: Actively decentres Eurocentric assumptions of individual autonomy by engaging African ontologies of collectivity, relationality, and embodiment. This disrupts universalist discourses that erase historically marginalised voices in SRHR policymaking.
3. **Structural transformation**: Interrogates how colonial legacies, neoliberal economic policies, and gendered power hierarchies perpetuate inequitable access to reproductive care. It explicitly links reproductive autonomy to broader struggles against racial capitalism, exploitation, and health system fragmentation.

## Results

The analysis of the selected court cases reveals the value of a decolonial and Africentric judicial approach to SRHR in Africa and the potential of the ARJ framework.

## CEHURD & Others v Attorney General SCCA 1/2013

In *CEHURD & Others v Attorney General SCCA 1/2013*,^56^ the Uganda Supreme Court advanced African contextual realities and positive African narratives in a case concerning maternal health, budget implications for the health sector, and the poor-quality care in government hospitals. The case arose from a ruling of the Constitutional Court wherein the appellants challenged certain acts and omissions of the government and staff in providing maternal health services in government hospitals/health facilities. These included the non-provision of basic indispensable maternal health commodities in government facilities; an inadequate number of midwives and doctors to provide maternal health services; inadequate budget allocation to the maternal health sector; and imprudent and unethical behaviour of health workers towards expectant mothers, which had resulted in the deaths of 800 women daily from preventable causes related to pregnancy and childbirth.^56^

The Court had to determine to what extent the political question doctrine – a legal principle whose origins date as far back as the early nineteenth century in the United States (US) Supreme Court – was applicable in Uganda. The doctrine had, until this case, been used by the state in Uganda to prevent judicial scrutiny of policy matters deemed preserved exclusively for the executive branch. The case provided an opportunity for the judicial branch to pronounce itself on the constitutional arrangement of the state’s three branches. The Supreme Court found that the political question doctrine had limited application in Uganda. It overturned decades of Ugandan precedent (and centuries of common law) that had shielded some executive decisions from judicial review. It held that:

*“the political question doctrine has limited application in Uganda’s current constitutional order and only extends to shield both the Executive arm of Government as well as Parliament from judicial scrutiny where either institution is properly exercising its mandate, duly vested in it by the Constitution”*.^56^

Further that:

*“If this Court were to uphold the respondent’s contentions to the effect that the political question in Uganda ousted the [Court’s] jurisdiction to inquire into the acts and omissions that the appellants had alleged were inconsistent with or in contravention of the Constitution, all acts and omissions of the Executive will be beyond judicial scrutiny”*.^56^

This decision all but sounded the death knell for the application of the political question doctrine in Uganda. First, the court noted that Uganda’s constitutional provisions were markedly different from those in the US, where the doctrine originates. The 1995 Constitution gives the right to any person who alleges that any act or omission of any person or authority contravenes the Constitution access to the Constitutional Court directly. Thereafter, the Constitutional Court is duty-bound to hear and determine the petition. In other words, unlike the US constitutional order enunciated in *Madison v Marbury,*^57^ no single provision of the Constitution is ring-fenced from interpretation and, by extension, judicial review or scrutiny, including those concerning policy formulation and implementation. The Supreme Court was at pains to stress that courts must be ready to use the law to serve social justice through a proactive, goal-oriented approach. In finding that actions or omissions of the Executive are immune from judicial scrutiny only if made pursuant to the Constitution, the Supreme Court effectively halted the application of the political question doctrine in Uganda. It directed that the matter be heard on its merits by the Constitution, resulting in a positive decision that required the government to increase expenditure to reduce maternal mortality; to stock drugs and related supplies; and to improve quality provision in public facilities.

The Constitutional Court heard the matter again in *CEHURD & Others v Attorney General* Constitutional Petition 16 of 2011^51^ and, in determining it on its merits, made several ground-breaking findings. First, it found that the right to health was justiciable in Uganda’s Constitution under Articles 8A, 39, and 45. Uganda’s Constitution omits a right-to-health provision in its substantive text. However, the National Objectives and Directive Principles of State Policy (NODPSP) oblige the state to ensure that “Ugandans enjoy rights and opportunities and access to … health services”.^58^ Article 8A, a recent amendment to the Constitution, requires the state to be governed in accordance with the NODPSP. This, the Constitutional Court pointed out, obliges the government to provide health and basic medical services to the people of Uganda.^50^ The Constitutional Court upheld the petition, holding that the failure to provide basic maternal health commodities was unconstitutional; the failure to implement well-known, affordable and effective approaches used to reduce preventable maternal death rendered the government liable for the deaths of the women who die as a result and was a violation of women’s rights guaranteed under the Constitution.

Among the remedies it granted the petitioners was an order requiring the government to prioritise and allocate sufficient funds in the national budget towards maternal health care; that government must ensure that all staff providing maternal health care is fully trained and all health centres are equipped within two years (2020–2022); that government must compile and submit to Parliament and the Court a full audit report on the status of maternal health in Uganda at the culmination of each of the next two financial years. The court required the government to submit a report detailing the progress and implementation of its orders.^59^

## CEHURD & 2 Others v Executive Director National Referral Hospital and Attorney General HCCS 212/2013

Another precedent-setting decision is *CEHURD & 2 Others v Executive Director of Mulago National Referral Hospital and Attorney General HCCS 212/2013*. This case is illustrative of the use of litigation to cause wide-ranging reform within the public health system on the continent. It also demonstrates the potential changes that can result from going beyond the black-letter law and abstract legal provisions to bring on-the-ground impact in the lives of African women and girls. A mother, one of the plaintiffs, delivered two babies but was discharged from the hospital managed by the first defendant with only one child. The hospital claimed that the second baby had died. To compound the parents’ grief, the parents were handed the wrong corpse of what the hospital staff said was the deceased child. DNA tests revealed no biological connection between the parents and the deceased child. The court had to determine whether the actions and inactions of the hospital staff were a violation of the rights of the child, the right to access health information and the right to health, the right to family, freedom from cruel, inhuman and degrading treatment and psychological torture.

It is commendable and worth mentioning that in this case, the judge heavily relied on and cited regional human rights instruments in reaching her decision. These include the African Charter on Human and Peoples’ Rights and the 1995 Constitution of the Republic of Uganda. This approach to judicial interpretation goes a long way in developing Africentric or African-centred jurisprudence that could wean us from depending on foreign precedent and the politics that might come with that. The judge was also alive to the need for the couple, as an “African couple,” to be accorded the opportunity to conduct the necessary rituals for their deceased child. She noted that the parents did not perform the ceremonies which “would have constituted a fundamental part of their healing process … and have been denied the opportunity to have closure in regard to their second baby [further] compounding their pain and subjecting them to more psychological torture … ”.

Further, the judge was alive to the systemic problems in the country that the case illustrated. For one, it was revealed that the mother received inadequate antenatal care, having visited a health facility just once during her pregnancy because she could not afford the costs of accessing health care. The WHO recommends a minimum of eight antenatal visits during pregnancy.^60^ As a result, the mother did not know that she carried twins. The judge also highlighted the shortage of health workers. In the instant case, the doctor who delivered the mother had to attend to twenty other patients that day. Yet, the daily recommended number is ten. The Court also raised concerns about respect and handling of the dead, especially babies, in medical facilities in Uganda.

Significantly, it was with the court’s remedies that the decision was groundbreaking for the provision of health care in Uganda. The Court found that the respondents’ conduct amounted to psychological torture and a violation of the right to health. Thus, it attempted to remedy the situation with systemic changes. First, it ordered the police to conclusively investigate the disappearance of the deceased child and file a report within six months. Second, the hospital was required to ensure that newborn babies, whether alive or stillborn, must be treated with respect and safety. Third, the hospital was required to make regular reports on the measures taken to fulfil the court’s orders. Fourth, the Court appointed the first plaintiff, a civil society organisation, as a quasi-ombudsperson over the hospital to monitor the implementation of its orders. Fifth, the courts ordered the hospital to pay for the parents’ psycho-social care and for the first plaintiff to ensure that they have access to the service. The parents were also awarded damages of UGX85M (approximately USD 23,500) as compensation.

## Igohozo v The Prosecutor

Until 2012, abortion in Rwanda was criminalised. In 2012, the country revised its penal code, allowing abortion strictly for cases of minors, rape, forced marriage, incest and instances where the pregnancy poses a health risk. The 2012 law also requires that women seeking an abortion get approval from a judge. Further, authorities proceeded to release 50 women who were jailed for having abortions after a personal pardon was issued by the country’s president.

In *Igohozo v The Prosecutor* RPA0787/15/HC/KIG, the Kigali High Court considered the case of a minor who had been raped and became pregnant. Her mother requested the court’s permission to terminate the pregnancy, but the court of first instance denied the request on several grounds: that it was unlikely a rape had occurred because no criminal charges had been raised against the alleged rapist; that the pregnancy could have resulted from causes other than rape; and that beyond the appellant’s suicide threat, there was no evidence to suggest that the pregnancy endangered her health, with her alternative justification deemed insufficient to warrant an abortion.

On appeal, the High Court of Kigali granted permission to terminate the pregnancy. The prosecution had argued that the victim had only been defiled, not raped, since defilement or statutory rape was not a recognised ground for termination. The Court rejected this argument, holding that both rape and defilement are forms of non-consensual sexual assault, distinguished only by the victim’s age. It further held that whether the pregnancy could have resulted from causes other than rape was immaterial, as minors are statutorily incapable of consenting to sex. Significantly, the Court accepted the victim’s reasons for seeking termination, *including her desire to return to school and to avoid social embarrassment*, as sufficient to justify the procedure. The decision thus reflects a small but important trend in expanding the grounds for safe and legal termination of pregnancy beyond purely medical indications, moving beyond the exceptions recognised under the Maputo Protocol.

## Mapingure v Minister of Home Affairs & Others (Civil Appeal SC 406 of 2012; SC 22 of 2014) [2014] ZWSC 22 (24 March 2014)

Zimbabwean activist and survivor, Mildred Mapingure, was attacked and raped in her home by robbers. She filed a criminal matter with the police and sought emergency treatment to prevent pregnancy and any sexually transmitted infection. Navigating the labyrinth of Zimbabwe’s administrative and legal system proved to be too much for Ms Mapingure. She was sent back and forth from the police to the prosecutor’s office to medical facilities for what ideally should have been a simple, humane medical process. As it turns out, the sexual assault left her pregnant, and by the time she obtained the authorisation to terminate the pregnancy, it was medically inadvisable to do so. She sued the government for damages in one of those rare decisions where an SRHR violation was settled through tort claims of negligence, thus expanding SRHR jurisprudence in this area. The court granted her damages for pain and suffering arising from the failure to prevent the pregnancy, finding that the police officers who refused to help her process a termination certificate were negligent in their duties. She was denied damages for the cost of maintaining the child because the court judged her responsible for not knowing what to do, implying that it was her fault for trusting what the authorities told her. Insufficient attention was paid to human rights norms and jurisprudence relevant to the matter.^35^ Although she was partially successful in her claim, Mapingure and her decision to take on a broken system are indicative of the need to “destabilise” the status quo on law, culture and politics in postcolonial Africa regarding safeguarding women’s rights generally and SRHR in particular.

## Discussion

The analysis of the selected court decisions reveals an appreciation within African jurisprudence towards a more holistic and context-specific approach to SRHR, aligning with the principles of the ARJ framework. The ARJ framework, rooted in the concept of Africentrism, emphasises the significance of addressing SRHR issues through an African-centred lens, considering the unique historical, cultural, and socioeconomic contexts of the continent.

In the context of SRHR litigation in Africa, Africentric Reproductive Justice is crucial as it offers a framework that is rooted in African perspectives, values, and solutions. It recognises that reproductive health issues are complex and deeply influenced by cultural, social, and historical factors unique to the African context.

The ARJ framework highlights the significance of amplifying positive African narratives on SRHR,^35^ countering the often negative narratives that portray African communities as passive recipients of aid or as lacking agency in addressing their own health needs.^35^ Amplifying positive African narratives on SRHR highlights the strengths and resilience of African communities, thereby empowering individuals and communities to take ownership of their reproductive health and rights through litigation.

Narrative change around reproductive justice has taken on renewed significance as anti-rights actors continue to galvanise opposition by claiming that their work safeguards African culture, norms and values.^61^ It is vital that this misleading discourse be challenged by amplifying the voices of those who seek to expand reproductive rights and reproductive justice, particularly those offering context-specific, community-grounded perspectives that reflect the lived realities of African people. The use of positive narratives here does not suggest telling uncritical or romanticised stories about the continent’s reproductive justice jurisprudence. Rather, it entails depicting the fuller picture: that Africa is not jurisprudentially desolate but is capable of providing leadership and examples, particularly at a time when anti-rights actors are advancing across the globe. Africa’s contribution and insights, drawn from its jurisprudence, can inspire, inform, and ignite similar litigation elsewhere in the world to safeguard reproductive rights and reproductive justice.^62^

The ARJ also advocates for the use of African-based institutions,^62^ such as the Community Court of Justice of the Economic Community of West African States, and the East African Court of Justice, amongst several others, to address reproductive justice issues. This is important because it ensures that legal and advocacy efforts are informed by decisions of African institutions, African legal frameworks, and cultural norms, leading to more culturally sensitive and effective interventions that ensure access to safe and legal abortion services in the region. The continent’s institutions have on several occasions blazed the trail on several contentious issues. As many scholars have pointed out, the African Union’s Maputo Protocol is the first, and so far, only binding human rights treaty enshrining a right to safe and legal abortion and post-abortion care in the world. With several institutions mandated to ensure the realisation of the rights guaranteed by the Maputo Protocol, it is imperative that a lot more resources and advocacy be dedicated to implementation. Currently, these mechanisms have been woefully underutilised in the pursuit of reproductive justice. Even so, they have a lot of potential. In *Centre for Reproductive Rights and Anor v Tanzania*, the African Committee of Experts on the Rights and Welfare of the Child (ACERWC), which is mandated to monitor the implementation of the African Charter on the Rights and Welfare of the Child, found that for African minors, the right to health encompasses the obligation of governments to provide comprehensive sexuality education, safe abortion and post-abortion care, among others.^63^ This decision has since been followed by a similar finding from the UN Human Rights Committee on Nicaragua's and Ecuador’s abortion restrictions for minors who are victims of rape.^64^ Thus, while challenges remain in ensuring the implementation of regional treaties at the national level, the work of the ACHPR and other African human rights bodies represents a significant step towards achieving reproductive justice across the continent.

The ARJ framework also emphasises the involvement of African-based civil society organisations,^64^ community groups, and other stakeholders in designing and implementing reproductive health programmes and policies. This inclusive approach ensures that interventions are responsive to the needs and priorities of African communities, leading to more sustainable and impactful outcomes.

One would be hard-pressed to find a decision seeking to expand reproductive rights and realise reproductive justice on the continent that has not been supported through logistical or technical expertise by indigenous and international civil society organisations. They have brought or defended suits in their own name – and where they have had to, supported litigating individuals to do so. In the *CEHURD & Ors v Mulago National Referral Hospital* case, the High Court appointed a civil society organisation as a quasi-ombudsperson to oversee the implementation of its decision. Today, civil society does not invoke the same passion and pride as it did a few decades ago. Organisations, especially those indigenous to the continent, have not proffered a coherent response to accusations of cultural imperialism, especially in the SRHR space. Nonetheless, it can be argued that none of the progress made over the last three decades, since the ICPD, would have been possible without civil society organising. However, civil society organisations must reinvent themselves – particularly concerning their funding and sustainability options – with a view towards securing their institutional and programmatic independence.

When compared to recent US jurisprudence on SRHR, the emerging African approach offers valuable insights. In *Whole Woman's Health v. Hellerstedt*, the US Supreme Court demonstrated a willingness to address the real-world impact of abortion regulations, emphasising how seemingly neutral laws can disproportionately affect marginalised communities. This approach aligns with the ARJ framework's emphasis on *context-specificity and intersectionality.* However, the subsequent *Dobbs v. Jackson Women's Health Organisation* decision, which overturned federal abortion rights protections, demonstrates the vulnerability of reproductive rights when solely reliant on legal precedent.

In contrast, the African cases examined indicate the capability and utility of incorporating reproductive justice principles into SRHR jurisprudence. These decisions address not only legal rights but systemic barriers, cultural contexts, and socioeconomic factors that influence reproductive health outcomes. Thus, the ARJ framework offers a more holistic and resilient approach to SRHR. This integration potentially provides a more stable foundation for reproductive justice that is less vulnerable to political shifts or changes in court composition.

Furthermore, the African cases demonstrate the imperative for recognising state responsibility in ensuring access to reproductive health services in contrast to the negative obligation of the now-overruled *Roe*. This emphasis on state obligation aligns with the ARJ framework's call for comprehensive, state-supported solutions to SRHR issues.

Moreover, court decisions like these establish important legal precedents that encourage African researchers and activists to consider reproductive litigations in the future, thereby contributing to the legal and jurisprudential development of reproductive rights in the African context. Beyond legal precedents, the cases expose systemic failures that prevent rape victims from accessing reproductive services to which they are legally entitled. Successful litigation in these areas can catalyse policy and legislative reforms to address these systemic issues, ultimately empowering and inspiring other victims to come forward and assert their rights.

The study also highlights the potential for the ARJ framework to guide the development of progressive SRHR policies and legislation in Africa. Through strategic litigation, the ARJ framework provides a roadmap for policy reforms that prioritise the needs and experiences of African women and girls.

However, the study also acknowledges the ongoing challenges in fully realising the goal of the ARJ framework, given the continuation of restrictive laws, deeply entrenched social and cultural barriers, and resource constraints across the continent. These challenges underscore the need for continued advocacy, community engagement, and capacity building to promote the wider adoption of the ARJ framework in African legal and policy contexts.

## Conclusion

The analysis of court decisions from various African nations reveals the value of a judicial approach that integrates both the reproductive rights and reproductive justice frameworks. They also highlight the necessity of addressing intersecting factors like race, class, gender, and socioeconomic status, which are integral to addressing long-standing injustices affecting SRHR in Africa. However, the current rights-centric approach often neglects the intricate web of unique African factors that influence SRHR experiences, perpetuating historical and structural injustices and impeding the complete realisation of SRHR in Africa.

The Africentric Reproductive Justice (ARJ) framework, grounded in decoloniality, offers a holistic and context-specific approach to addressing the unique SRHR challenges on the African continent. This framework emphasises several critical elements that facilitate the emergence from a choice-centric approach to a more comprehensive reproductive justice paradigm.

Firstly, it highlights the importance of amplifying positive African narratives on SRHR by showcasing African-led initiatives and successes in advancing reproductive rights. This counters the often negative narratives that portray African communities as passive recipients of aid, instead empowering individuals and communities to take ownership of their reproductive health and rights.

Secondly, the ARJ framework leverages African-based judicial institutions, with case reviews demonstrating that African courts are increasingly willing to balance competing rights and interests in a manner sensitive to the cultural and religious diversity of African societies. By utilising and strengthening these institutions, the ARJ framework ensures that legal decisions are grounded in African realities and values.

Thirdly, involving African civil society organisations is crucial. The ARJ framework recognises the vital role of local civil society in advocating for and implementing SRHR policies. These organisations provide invaluable insights into community needs and can effectively bridge the gap between policy and practice.

Lastly, the ARJ framework focuses on empowering African individuals through capacity building and education. This aims to equip African individuals with the knowledge and skills needed to contribute meaningfully to reproductive justice efforts, which is critical for sustainable, long-term change.

These elements are instrumental in shifting from a narrow focus on reproductive choice to a more comprehensive reproductive justice approach. They address the limitations of the current rights-centric approach, which often neglects the intricate web of unique African factors that influence SRHR experiences and perpetuate historical and structural injustices.

While the integration of substantive equality into reproductive rights frameworks is a step in the right direction, it falls short of the comprehensive approach needed to fully address the complexities of SRHR issues in Africa. Reproductive justice encompasses these elements and goes further by addressing the historical and structural injustices that impede the full realisation of SRHR. This is important because the challenges surrounding SRHR in Africa are deeply rooted in systemic and structural issues.

To effectively implement the ARJ framework in strategic litigation, this study recommends fostering collaboration between legal practitioners, civil society organisations, and SRHR advocates; developing a network of African legal experts specialising in SRHR; promoting the use of African human rights instruments; engaging in capacity-building initiatives; and advocating for the inclusion of the ARJ framework in legal education curricula.

In conclusion, achieving genuine reproductive justice necessitates moving beyond the “right to choice”. It requires deconstructing enduring structural barriers and addressing historical injustices perpetuated by colonial legacies. Judicial decisions with the potential to advance reproductive justice must be contextual, multifaceted, and culturally sensitive to Africa's SRHR needs. The Africentric Reproductive Justice framework, rooted in decolonial principles and embodying the key elements discussed, presents a potential pathway towards achieving equitable SRHR for all individuals on the continent. This approach not only seeks legal recognition of rights but also recognises the unique context and complexities of the African environment, offering a more resilient and sustainable approach for advancing reproductive justice in Africa and potentially in other parts of the world.

## Author contributions

Conceptualisation: MM, JO, NM. Writing – original draft: JO. Writing – review & editing: MM, JO, NM.

## Footnotes

* In *State v Makwanyane*, 1995 (3) SA 391 (CC) paras 237, 260, the South African Constitutional Court held that Ubuntu, though not explicit in the substantive provisions, permeates the Constitution and the Bill of Rights; The Nigerian Supreme Court has also adopted constitutional and extra-constitutional principles under rubrics such as “peace, order, and good government, fundamental objectives and directive principles of state policy, constitutional supremacy and cooperative federalism”.

† Common popular and academic parlance often uses the term “Afrocentrism” to refer to the placing of African ideals at the center of any analysis that involves African culture and behavior. We prefer to use the prefix “Afri” because we believe it to be more appropriate in delineating and speaking to the experiences of Africans on the continent.
